# Inflammatory Response Mechanisms of the Dentine–Pulp Complex and the Periapical Tissues

**DOI:** 10.3390/ijms22031480

**Published:** 2021-02-02

**Authors:** Kerstin M. Galler, Manuel Weber, Yüksel Korkmaz, Matthias Widbiller, Markus Feuerer

**Affiliations:** 1 Department of Conservative Dentistry and Periodontology, University Hospital Regensburg, 93093 Regensburg, Germany; matthias.widbiller@ukr.de; 2 Department of Oral and Maxillofacial Surgery, Friedrich-Alexander University Erlangen-Nürnberg, 91054 Erlangen, Germany; manuel.weber@uk-erlangen.de; 3 Department of Periodontology and Operative Dentistry, University of Mainz, 55131 Mainz, Germany; yueksel.korkmaz@unimedizin-mainz.de; 4 Department for Immunology, University Hospital Regensburg, 93053 Regensburg, Germany; markus.feuerer@ukr.de; 5 Regensburg Center for Interventional Immunology (RCI), University Hospital Regensburg, 93053 Regensburg, Germany

**Keywords:** dental pulp, odontoblast, tertiary dentine, immune response, carious lesion, pulpitis

## Abstract

The macroscopic and microscopic anatomy of the oral cavity is complex and unique in the human body. Soft-tissue structures are in close interaction with mineralized bone, but also dentine, cementum and enamel of our teeth. These are exposed to intense mechanical and chemical stress as well as to dense microbiologic colonization. Teeth are susceptible to damage, most commonly to caries, where microorganisms from the oral cavity degrade the mineralized tissues of enamel and dentine and invade the soft connective tissue at the core, the dental pulp. However, the pulp is well-equipped to sense and fend off bacteria and their products and mounts various and intricate defense mechanisms. The front rank is formed by a layer of odontoblasts, which line the pulp chamber towards the dentine. These highly specialized cells not only form mineralized tissue but exert important functions as barrier cells. They recognize pathogens early in the process, secrete antibacterial compounds and neutralize bacterial toxins, initiate the immune response and alert other key players of the host defense. As bacteria get closer to the pulp, additional cell types of the pulp, including fibroblasts, stem and immune cells, but also vascular and neuronal networks, contribute with a variety of distinct defense mechanisms, and inflammatory response mechanisms are critical for tissue homeostasis. Still, without therapeutic intervention, a deep carious lesion may lead to tissue necrosis, which allows bacteria to populate the root canal system and invade the periradicular bone via the apical foramen at the root tip. The periodontal tissues and alveolar bone react to the insult with an inflammatory response, most commonly by the formation of an apical granuloma. Healing can occur after pathogen removal, which is achieved by disinfection and obturation of the pulp space by root canal treatment. This review highlights the various mechanisms of pathogen recognition and defense of dental pulp cells and periradicular tissues, explains the different cell types involved in the immune response and discusses the mechanisms of healing and repair, pointing out the close links between inflammation and regeneration as well as between inflammation and potential malignant transformation.

## 1. Introduction

**Figure 1:**
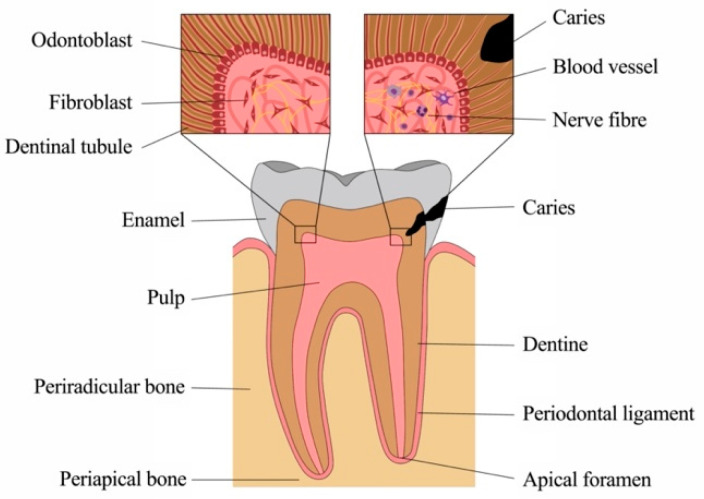
Anatomy and physiology of the tooth.

Although small, teeth are complex structures composed of several components with unique architectural characteristics and functions (Figure 1). Crown and root are made of different mineralized tissues, namely enamel, dentine and cementum, encasing a soft tissue, the dental pulp. The root anchors the tooth to the surrounding bony tissue via short, tendon-like fibers called the periodontal ligament, which insert both into root cementum and bone.

Teeth are prone to damage, mostly by caries, periodontal disease and trauma. In all these cases, microorganisms lead to infection and inflammation. Advanced methods and materials are available in dental medicine to date, where most therapies aim at the replacement of damaged or lost structures. Yet, oral tissues possess multiple means of pathogen recognition and defense, which are currently investigated and understood in increasing detail. An in-depth understanding of immune reactions of dental tissues, of cellular and molecular key players, as well as of temporospatial patterns of defense will enable approaches to improve diagnostics as well as treatment strategies, which can be more targeted, less invasive and aimed at tissue healing and regeneration rather than replacement.

### 1.1. Anatomy and Physiology of Sound Dental Tissues

#### 1.1.1. Physiology of the Dentine-Pulp Complex

The craniofacial tissues are mainly derived from cells of the cranial neural crest. These cells develop in the dorsal region of the neural tube and then migrate into the 1st–fourth pharyngeal arch [1]. In the dental pulp, cranial neural crest-derived cells of the pulpal neurons play an important role in the regeneration of mesenchymal pulp cells and odontoblasts [2]. Most parts of the teeth are formed by cranial neural crest cells, namely dentine, cementum, periodontal ligament and the pulpal tissue, with the exemption of blood vessels and the enamel [3].

**Figure 2:**
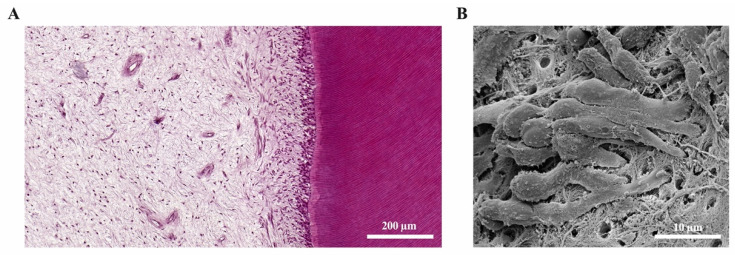
The dental pulp. (**A**) Histology of the dentine-pulp complex (own collection). (**B**) Odontoblast layer depicted by scanning electron microscopy (modified from [12]).

The dental pulp and the surrounding dentine (Figure 2A) form a unity, both developmentally and structurally. The pulp is made of mesenchymal soft connective tissue; it extends from a central chamber within the tooth crown into one or several root canals to the root apex. During tooth development and eruption, the presence of functional pulp tissue is a prerequisite for the completion of root formation. The pulp is lined with a layer of highly specialized cells, the odontoblasts (Figure 2B). These post-mitotic, polarized cells secrete a collagenous matrix, which later mineralizes to form dentine. This formative process occurs physiologically and continuously, not only during tooth development (primary dentine) but also later in life (secondary dentine). Each cell leaves a process behind, which becomes embedded in the mineralized tissue, giving dentine its tubular structure. While its composition is similar to that of bone, and odontoblasts share many characteristics with the osteoblasts, there are a number of distinct differences. Odontoblasts secrete dentine in a directional manner, and the cell bodies are not enclosed in the mineralized tissue. Thus, there is no physiological remodeling and replacement of dentine. Due to their origin from neural crest-derived ectomesenchyme and their unique localization, odontoblasts feature many more characteristics than just those of mineralizing cells. Shielded by enamel and dentine, they are the first line of cells to get in contact with toxins and compounds of oral bacteria once the mineralized matrices have started to break down, with caries being the most prevalent cause, followed by dental trauma. Thus, odontoblasts play a central role as mediators of both inflammation and repair processes [4,5,6]. Beneath the odontoblast layer and an adjacent cell-free zone, pulp fibroblasts populate an extracellular matrix mainly made of collagen type I and III [7]. The pulpal core is crossed by vascular and neuronal networks, which enter the tooth through the alveolar bone via the apical foramen. An accumulation of nerve fibers (plexus of Raschkow) can be found beneath the layer of odontoblasts; these follow the odontoblast processes into the dentinal tubules, making dentine and innervated tissue. It is assumed that there is either intimate crosstalk between the odontoblasts and the nerve fibers or that odontoblasts themselves participate in the transmission of external stimuli [8]. The majority of the pulpal tissue is composed of pulp fibroblasts. Stem cells, which are present in dental pulp in the perivascular niche, exhibit both mesenchymal and neural characteristics due to their origin from ectoderm. The pulp of healthy teeth is furthermore equipped with a variety of cells of the immune system. A recent study that investigated immune cells in healthy human pulp confirmed previous reports [9,10] and demonstrated that leukocytes (cluster of differentiation 45-positive/CD45+ cells) are present but contribute less than 1% to the total cell population [11]. Among these, granulocytes/neutrophils (CD16+ CD15+ CD14−) were found to be the major subpopulation, followed by CD3+ T lymphocytes, CD14+ monocytes and dendritic cells, whereas minor subpopulations included natural killer (NK) cells, B cells and regulatory T cells (Tregs) [11]. These immune cells, in correspondence with odontoblasts, pulp fibroblasts and pulpal stem cells, are essential for the initiation of immunological responses of the dental pulp to oral microorganisms.

#### 1.1.2. Physiology of the Apical Periodontium and Periradicular Tissues

Similar to the dental tissues, the skeleton of the face and a large majority of craniofacial connective tissue is derived exclusively from cells of the cranial neural crest [1,3]. Within the facial bones, in particular the alveolar bone, teeth are anchored via the periodontal ligament as part of the periodontium, which attaches the root cementum to the surrounding alveolar bone. The fibers of the periodontal ligament absorb and transmit forces between teeth and bone during the masticatory function. Alveolar bone undergoes constant physiologic remodeling in response to occlusal forces, which influence the number, density and alignment of trabeculae inside the bone.

Apical periodontitis is caused by an infection of the dental pulp that extends to the root pulp and leads to its necrosis [13]. The loss of the pulp tissue accounts for the loss of immune function, which allows microorganisms to access the alveolar bone via the root canal system. Periapical lesions can appear clinically as apical granulomas or radicular cysts and are both of inflammatory origin. In this context, the same etiology (necrosis of the pulp) leads to different clinical pathologies (apical granulomas and radicular cysts). Radicular cysts can grow to large extents and lead to the destruction of the periradicular periodontal tissue and the surrounding jaw bone [14]. In severe cases, even continuity resections of the mandible might be necessary for proper treatment of radicular cysts.

The epithelium of radicular cysts is most likely derived from the epithelial cell rests of Malassez (ERM). The ERM cells are a physiologic component of the periodontal ligament and originate from Hertwig’s epithelial root sheath (HERS), which governs dental root formation during embryologic development [15]. As the HERS undergoes an incomplete involution after the completion of root development, vital ERM cells remain in the periodontal ligament of the adult organism [15,16]. These ERM cells are critical for the physiology of periradicular tissues, as they keep their characteristics of epithelial cells despite the fact that they are embedded in a mesenchymal matrix. ERM cells are critical for periodontal ligament homeostasis and maintenance of the periodontal space, and they are involved in the prevention of ankylosis and root resorption [15].

## 2. Inflammatory Response of the Dental Pulp

**Figure 3:**
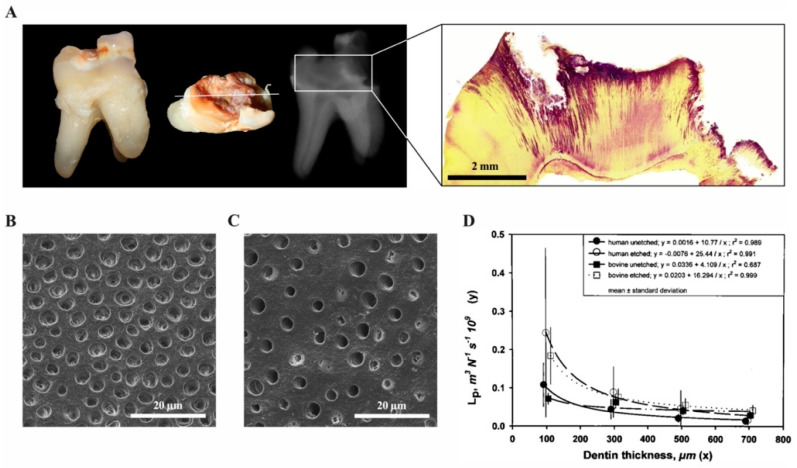
The carious process (own collection). (**A**) Extracted tooth with a deep carious lesion: clinical, radiographic and histologic appearance. The carious process has degraded enamel and dentine, which can be seen as radiolucent areas on the radiograph. After Brown and Brenn staining of the same tooth, bacteria are visible in the dentinal tubules (purple). (**B**) Dentine surface with dentinal tubules in a deep lesion close to the pulp and (**C**) a shallow cavity. (**D**) Permeability of dentine represented by hydraulic conductance L_p_ as a function of thickness for human and bovine dentin. Points with vertical lines represent the means and SD of the original data; lines represent the regressions of y vs. 1/x. (modified from [18]).

Inflammatory reactions in the dental pulp have been described and extensively studied in response to a variety of insults such as caries, periodontal disease, operative procedures and dental trauma. As the oral cavity is heavily populated by microorganisms, the dental pulp is able to mount innate and later adaptive immune responses to inactivate and fight bacteria and their components that gain access during the carious process. In healthy teeth, a protective layer of enamel prevents the invasion of microorganisms through dentine and towards the pulp. Additionally, a continuous outward flow of dentinal fluid flushes out sporadically migrating bacteria. Carious demineralization of enamel caused by acidic metabolites of specific bacterial populations leads to disruption of the barrier, to cavitation and to degradation of dentine by Gram-positive bacteria, including streptococci, lactobacilli and actinomyces that largely dominate the microflora [17]. As both the diameter and the density of tubules that allow bacterial penetration increases with closer proximity to the pulp, this process accelerates with increasing depth of the lesion (Figure 3) [18].

### 2.1. Recognition of Pathogens and Signal Transmission

Proliferating and metabolically active bacteria release components, which elicit antibacterial, immune and inflammatory reactions within the pulp tissue, where noticeable histological changes can be observed before the lesion even reaches into dentine. Despite the immense variety of bacteria, groups of pathogens share similar structures that are essential for their survival; these signature molecules called pathogen-associated molecular patterns (PAMPs) enable the host cells to recognize the threat via pattern-recognition receptors (PRRs). Whereas different families of PRRs have been identified, these germ-line encoded receptors recognize a number of conserved microbial molecules and are expressed in a wide variety of host cells. Differences exist regarding the evoked signaling cascades and responses as well as their expression levels in different tissues. Thus, the initial invasion of microbial components and toxins activates innate immunity, which is not specific to antigens, but to bacteria or their products, and initiates an attack and elimination by phagocytotic killing. Due to the unique location of bacteria in carious lesions, phagocytosis will not take place until the carious front has actually reached the pulp. Odontoblasts, pulp fibroblasts, leukocytes and pulpal stem cells express PRRs [19,20,21,22], in particular Toll-like (TLR) and NOD-like receptors (NLR) [23], which indicates that the dental pulp is equipped to sense a wide variety of pathogens that could invade the pulp chamber. These receptors recognize, among others, triacetylated lipopeptides (TLR1/2), diacetylated lipopeptides and lipoteichoic acid (LTA) (TLR2/6), viral dsRNA (TLR3), lipopolysaccharides (TLR4), flagellin (TLR5) and unmethylated CpG motif-containing DNA (TLR9) [24]. Carious lesions are predominantly caused by Gram-positive bacteria; their cell wall component LTA is recognized by TLR2 engagement. Expression of TLR2 is upregulated beneath carious lesions, suggesting an amplification of the response [25]. Furthermore, the NLR receptor NOD2 is expressed by most cells in healthy dental pulp, including odontoblasts, pulp fibroblasts and perivascular cells [23]. Antigen binding to TLR2 and the cytosolic NOD2 leads to the activation of nuclear factor-κB (NF-κB) and p38 mitogen-activated protein kinase (MPK) signaling, resulting in the production of proinflammatory cytokines and chemokines, which can recruit dendritic cells [5,19,25] and other immune cells towards the dentine-pulp interface beneath the carious lesion to neutralize bacterial toxins [26,27], and also leading to an inhibition of dentinogenesis if the insult is severe [21,28].

### 2.2. The Role of Odontoblasts

**Figure 4:**
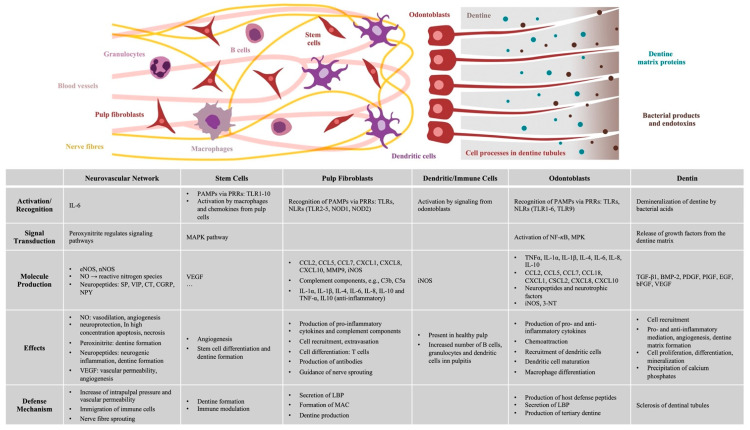
Outline of all structural and cellular components involved in the immune defense of the dental pulp.

Due to their anatomic position in the periphery of the pulp with their cellular processes extending into the dentinal tubules, the odontoblasts form the first line of detection as well as defense (Figure 4). Thus, they fulfill barrier functions shared with other barrier cells, such as epithelial cells of skin and gut mucosa [19,20]. Odontoblasts constitutively express the above-mentioned Toll-like and NOD-like receptors [23], including TLR1-6 and TLR9 [19]. Upon receptor binding, odontoblasts resort to a repertoire of defense mechanisms. They secrete several antibacterial products, also via their cellular processes that are embedded in the dentine matrix, among them cationic host defense peptides (HDPs) such as beta-defensins (BDs). These disrupt the membrane integrity of microorganisms, thus exerting a broad spectrum of antimicrobial activity [29]. BDs are expressed by epithelial and immune cells, either constitutively or induced by microorganisms [30]. Moreover, host defense peptides exhibit immunomodulatory activities [31,32], among others the induction of pro-inflammatory cytokine production in immune cells (TNFα, interleukin 1α/IL-1α, IL-6, IL-8, chemokine ligand 18/CCL18) [33], chemoattraction [34], the promotion of dendritic cell maturation [35] as well as macrophage differentiation [36]. The expression of human BDs 1 and 2 by odontoblasts in healthy pulps has been demonstrated [37]. Furthermore, stimulation of pulp cells with BD2 resulted in an upregulation of IL-6, IL-8 and cytosolic phospholipase-A-2, confirming the immunomodulatory function of beta-defensins in human pulp [38]. BD3, which disrupts cell wall biosynthesis by binding lipid-II-rich regions of the cell wall [39], is induced in dental pulp by heat as well as bacterial lipopolysaccharides. This beta-defensin appeared to be more effective against a mixed biofilm of bacteria commonly found in infected root canals (*Actinomyces naeslundii*, *Lactobacillus salivarius*, *Streptococcus mutans*, *Enterococcus faecalis*) than treatment with chlorhexidine in an in vitro study [40]. However, the bactericidal activity of defensins in vitro does not necessarily reflect functional activity in vivo, as it requires high amounts [29], whereas their various immunomodulatory effects take effect already at low concentrations.

Besides the odontoblasts, pulpal dendritic cells (DC), which are localized in close vicinity (Figure 4), but as well in the perivascular regions, play an important role in immunosurveillance and capture of foreign antigens [41,42]. Corresponding to the progression of carious lesions within the dentine, DCs accumulate at the dentine-pulp-interface [43]. Odontoblasts and dendritic cells cooperatively induce the pulpal responses by the expression of cytokines and prostaglandins and the sequential attraction of inflammatory cells, namely T cells, macrophages, neutrophils and B cells, and an increased CD4/CD8 ratio of T-lymphocytes [4,44].

Protective measures include the production of the acute-phase protein LBP (lipopoly-saccharide-binding protein), which neutralizes bacterial cell wall components and can attenuate the immune response by inhibiting the production of proinflammatory cytokines [6]. Challenged with bacterial toxins, odontoblasts furthermore produce vascular endothelial growth factor (VEGF), a potent inducer of vascular permeability and vasculogenesis [45].

### 2.3. Immunocompetence of Pulp Fibroblasts

Whereas Gram-positive bacteria greatly dominate the microflora in initial and moderate carious lesions, the proportion of Gram-negative anaerobic bacteria increases in deeper lesions [17,46,47] and infiltration of the dental pulp with inflammatory cells becomes evident [46,48]. As microorganisms start to invade the pulp, the destruction of the odontoblast layer can be observed, and the subjacent pulp fibroblasts are activated and participate in the host response (Figure 4). Similar to odontoblasts, they sense pathogens as they express several TLRs (TLR 2–5) as well as NOD1 and NOD2 [5,21,49]. In response to PAMPs, they produce proinflammatory cytokines and chemokines, including chemokine (C-C motif) ligand 2 (CCL2), CCL5, CCL7, chemokine (C-X-C motif) ligand 8 (CXCL 8) and CXCL10 [5,21]. These cytokines expressed by odontoblasts and later fibroblasts regulate immune cell recruitment, extravasation, cell activation, differentiation and influence antibody production.

Only recently, the role of pulp fibroblasts in the process of defense and regeneration has been explored in more detail. Pulp fibroblasts—as a nonimmune cell type—produce all components of the complement system [50]. Whereas these small proteins of the complement system are mostly synthesized in the liver, local production of complement components can enhance the inflammatory response but also guide healing. Furthermore, pulp fibroblasts are capable of producing the complement components to form the membrane attack complex (MAC), which is effective against cariogenic bacteria [51]. Bacterial elimination by complement-mediated phagocytosis requires opsonization with the complement C3b protein, which is recognized by phagocytic cell CR1 receptors for subsequent intracellular destruction. Pulp fibroblasts mediate this process by producing the complement C3b protein. On a different account, stimulation with bacterial toxins enables pulp fibroblasts to guide nerve sprouting during pulp regeneration through complement system activation and the production of neurotrophic factors [52,53]. These findings highlight the crucial role of fibroblasts in the regulation of inflammation within the dental pulp.

### 2.4. Inflammatory Signaling Molecules and Accumulation of Immune Cells

Upon pathogen recognition, odontoblasts, immune cells and later pulp fibroblasts produce a multitude of signaling molecules to orchestrate the immune response. Initially, odontoblasts produce chemokines, in particular, CCL2, CXCL1, CXCL2, CXCL8 and CXCL10 [21,28], to attract dendritic cells and other immune cells. The various cytokines within the pulp are now well-characterized, including IL-1α, IL-1β, IL-4, IL-6, IL-8, IL-10 and tumor necrosis factor α (TNF-α), which are known to control many aspects of the inflammatory response, dependent on their levels and profiles [54]. In experimentally induced pulpitis in rats, IL6, IL-1β, TNF-α, CCL2, CXCL1, CXCL2, also MMP9 and inducible nitric oxide synthetase (iNOS) gene expression were significantly upregulated only 3 h after stimulation with lipopolysaccharide (LPS), and a significant local increase of leukocytes was evident 6 h later [28]. IL-6, a cytokine expressed by odontoblasts and immune cells, is strongly upregulated in the inflamed pulp [55,56]. Among other functions, it plays a critical role in the differentiation of T cells, namely the T helper Th17 phenotype, promotes the secretion of LBP and increases vascular permeability [57], which results in the formation of edema [55]. Another important player, IL-10, acts as an anti-inflammatory by decreasing the production of pro-inflammatory cytokines, in particular IL-6 and CXCL8, thus suppressing the immune response and limiting tissue damage [58]. It also inhibits Th1 and Th2 immune responses but promotes the differentiation of Tregs, in part by a positive regulatory loop for IL-10 induction [59,60]. IL-10 is upregulated in inflamed pulps and also in odontoblast-like cells in vitro upon TLR2 engagement [55], suggesting that odontoblasts are capable not only of initiating the pulp‘s response to dentine-invading bacteria but also of limiting its intensity. Moreover, cytokines, neuropeptides and neurotrophic factors, which are described in more detail below, are expressed by odontoblasts [61], potentially due to their close association with the subjacent neuronal network and due to their origin from the CNC.

Multifaceted functions in the context of pulpal inflammation have been described for nitric oxide (NO), which is synthesized by the enzymes called NO synthases [62,63]. These enzymes catalyze the production of NO via oxidation of L-arginine to citrulline [64,65]. They are produced by neuronal cells (called nNOS) [66,67], genuine immune cells (iNOS) [68,69] and endothelial cells (eNOS) [70,71]. The effect of NO depends on its concentrations in cellular and subcellular compartments [72,73]. While the complex mechanisms of NO action must be elucidated yet, it appears that low concentrations of NO, which act within seconds to minutes, promote vasodilation, angiogenesis and tissue homeostasis; medium concentrations may be involved in neuroprotection, and high concentrations, which act within hours to days exert nitrosative stress and induce apoptosis, necrosis and tissue damage [74,75,76,77]. NO serves both as a biological effector and intracellular messenger and is a versatile endogenous molecule, which regulates different aspects of physiological and pathological events in the dental pulp, including cell proliferation [78], vasodilatation [79], neuromodulation [80] and odontoblastic differentiation [78,80]. In dental pulp, nerve fibers are a source of nNOS, and endothelial cells produce eNOS; both have been identified in odontoblasts [80,81]. In resting cells, iNOS is not expressed, but it is expressed in cells during physiological activity [82,83] and during pre- and postnatal development [84,85]. A weak expression of iNOS has been detected in healthy odontoblasts [86]. Interestingly, the expression of iNOS was observed only in a subpopulation of healthy odontoblasts, but not in all odontoblasts, which may indicate a physiological activation state (differentiation) of this subpopulation within the odontoblast layer under physiological conditions [80,87]. These results suggest that the state of differentiation of cells within the odontoblast layer may be heterogeneous.

During inflammation, high concentrations of NO produced by iNOS due to effects of inflammatory mediators such as IL-1, IL-6, TNF-α, interferon γ (IFN-γ), and LPS are unstable and oxidized to reactive nitrogen species (RNS) [88,89]. In higher NO concentrations, superoxide (O_2_^−^) interacts with NO to generate peroxynitrite (OONO^−^) in toxic concentrations [88,90]. Under physiological conditions, OONO^−^ are generated in low concentrations and regulate various signaling pathways. An eventual cellular nitrosative damage by OONO^−^ is balanced by endogenous antioxidant defenses [90,91]. During inflammation, higher concentrations of OONO^−^ inhibit the activity of mitochondrial enzymes and numerous transcription factors, thus producing long-term and highly toxic cellular effects [90,92]. In irreversible inflammation of the human dental pulp following deep dentine caries, a strongly increased expression of iNOS and 3-nitrotyrosine (3-NT), an indicator of cell damage during inflammation, was detected in odontoblasts [87]. The higher expression of the peroxynitrite-marker 3-NT in odontoblasts of the inflamed pulp suggests that oxidative phosphorylation and transcriptional regulation of dentine matrix formation may be disturbed by a higher concentration of peroxynitrite.

While immune cells are part of the healthy pulp (Figure 4), a significant increase of leukocytes was observed in rat pulpal tissue 9 h after the exposure to LPS. Within this population, percentages of B cells as well as myeloid cells, in particular granulocytes and dendritic cells, were increased; at that time point, percentages of T cells and NK cells remained unchanged [28].

### 2.5. Tertiary Dentine Formation

An essential feature of pulpal defense and wound healing is the formation of tertiary dentine to create a mineralized barrier and separate the pulp from the site of injury and bacterial invasion [93]. Many aspects of similarity have been demonstrated between primary and tertiary dentinogenesis, where both processes are signaled and driven by a similar array of bioactive molecules [94,95]. Tertiary dentine is formed either as reactionary or reparative, which must be distinguished from one another, as they arise from two different populations of cells, and thus their genesis and nature are distinct. Reactionary dentine formation is the result of a locally increased secretory activity of odontoblasts in response to mild stimulation [94,96]. This is mainly attributed to the presence of transforming growth factor β1 (TGF-β1), a potent stimulator of odontoblast differentiation and matrix secretion [94]. In carious lesions, the demineralization of dentine induced by bacterial acids results in subsequent solubilization of bioactive molecules, in particular TGF-β1, the most abundant non-collagenous protein found in the dentine matrix [97,98]. Additionally, a gradual occlusion of the dentinal tubules by centripetal deposition of calcium phosphate crystals at the mineralization front and along the odontoblast processes can be observed, leading to sclerosis and thus a decreased permeability of dentine [94,99,100].

In contrast, reparative dentinogenesis is a more complex biological process. Stronger stimuli will lead to the death of the odontoblasts, but other cells will deposit mineral, either pulp fibroblasts or cells that are recruited from the pool of stem cells. Reparative dentine is atubular, characterized by an amorphous structure and entrapped cells. It remains unclear whether osteodentine formation is a result of this cell source, which is not comprised of original odontoblasts, or to the intensity of stimulation, which leads to hasty deposition of a mineralized tissue that is less organized.

### 2.6. Vascular and Neuronal Networks

Not only cellular interactions but also an intricate neurovascular interplay is crucial during the inflammatory response of the dental pulp to microorganisms. Upon stimulation, sensory nerve fibers not only transmit pain but are able to induce neurogenic inflammation by secretion of neuropeptides, in particular substance P (SP), vasoactive intestinal polypeptide (VIP), calcitonin (CT), calcitonin gene-related peptide (CGRP) and neuropeptide Y (NPY) [101]. The release of these compounds results in vasodilation and increased vascular permeability, furthermore to the recruitment and activation of immune cells. It has been found that the levels of SP are significantly elevated in carious compared to sound teeth [102]. Neuropeptides may furthermore promote tertiary dentine formation by increasing the expression of bone morphogenetic protein 2 (BMP-2), which stimulates the secretory activity of the odontoblast [101]. The inflammatory process and an injury of sensory nerve fibers trigger the sprouting of terminal branches of neurons into the remaining healthy pulpal tissue [103]. Interestingly, these newly formed branches can amplify the inflammatory response by increased secretion of neuropeptides [104,105]. Distinct differences concerning healing and repair were demonstrated in a rat model after pulp exposure in innervated compared to denervated molars, where less tissue survival and enhanced damage due to necrosis was observed in denervated teeth, which appear to be less fit for defense [104].

During the initial phase of caries progression, the above-mentioned protective outward flow of liquid in the dentinal tubules can be increased as a result of elevated intrapulpal pressure after the release of neuropeptides by intra-pulpal sensory afferent nerve fibers [106,107]. Consequently, the number of invading microorganisms is significantly higher in nonvital compared to vital teeth [108], highlighting the well-coordinated interplay of neuronal and vascular networks within the dental pulp.

Trigeminal nerve endings, pulpal nerve fibers and odontoblasts are nNOS-positive [80,109]; however, the nitrergic system shifts back to the initial state even if pulpal inflammation persists [110]. Whereas the actions of nNOS in dental pulp are poorly understood, it appears to be involved in the regulation of pulpal blood vessels (vasodilatation) and neuromodulation in health and disease [80,110]. It was described that nNOS regulates sensory nerve-mediated neurogenic inflammation [111] and that nNOS inhibitors are able to inhibit CGRP-induced vasodilation [112]. A decrease in mRNA levels of VIP in nNOS-knockout mice indicating a relation for expression between nNOS and VIP [113]. In addition, NO upregulates migraine-related CGRP in neurons of the trigeminal ganglion [114]. Together, these findings suggest that NO generated by activation of nNOS in pulpal nerve fibers may regulate the release of neuropeptides, which may also trigger neurogenic inflammation in the dental pulp. In the blood vessels of the dental pulp NO-sensitive, the enzyme heterodimeric soluble guanylyl cyclase (sGC) and cyclic guanosine monophosphate (cGMP) were detected [80]. In addition to the production of NO by the activity of eNOS in endothelial cells of the dental pulp [80,81], this raises the possibility that nNOS-derived NO can also diffuse into adjacent vascular smooth muscle cells to potentiate vasodilation via the NO-cGMP pathway in the dental pulp.

Physiological amounts of tetrahydrobiopterin (BH4) are essential for the catalytic activity and stabilization of eNOS in its homodimeric form (coupled eNOS), in which eNOS generates biological NO [63,71]. In inflammation, NADPH oxidases are strongly active in the vascular wall and generate higher concentrations of O_2_^−^ [70,115]. In inflamed vascular walls, the product of NADPH oxidases O_2_^−^ and eNOS-derived NO rapidly form ONOO^−^, which strongly oxidizes BH4 to the inactive BH3 and BH2 in the structure of eNOS [116,117]. As a result, eNOS loses its homodimeric form and transforms to the uncoupled form, in which eNOS generates O_2_^−^ instead of NO [63,118]. In the inflamed dental pulp, a higher expression of 3-NT was described [87]. Based on these findings, it may be suggested that, in inflamed dental pulp, eNOS is uncoupled due to higher formation of ONOO^−^ and may generate O_2_^−^ instead of NO.

### 2.7. The Role of Signaling Molecules within the Dentine Matrix

During tooth development, terminally differentiated odontoblasts start to produce dentine. They first lay down a collagenous matrix, which later calcifies to form mineralized dentine. In addition, the odontoblasts secrete various signaling molecules [119,120,121], which remain embedded within dentine, bound to extracellular matrix components such as proteoglycans [122,123] and glycoproteins [124] but also various types of collagen [125,126]. These bioactive compounds can be activated by dentine demineralization in a carious lesion [127] and contribute to the immune response. Signaling molecules that are present within the dentine matrix include mineralization-associated proteins, growth and differentiation factors, cytokines and neurotrophic factors, as well as complement components [128,129]. Growth factors that are bound in human dentine and released after demineralization include TGF-β1 as the most abundant, in addition, BMP-2, platelet-derived growth factor (PDGF), placenta growth factor (PIGF) and epidermal growth factor (EGF), but also angiogenic factors such as basic fibroblast growth factor (bFGF) and VEGF [119,120,130]. These molecules play a role in the immune response as pro- or anti-inflammatory mediators; they exert chemotactic effects and recruit cells, promote angiogenesis and stimulate the proliferation and differentiation of progenitor cells and influence mineralization, even at minute concentrations [97,131,132,133]. Accumulating evidence suggests a fundamental role for dentine matrix proteins during the inflammatory response, which becomes even more decisive in deep carious lesions [134,135].

## 3. Inflammatory Responses in the Periapical Bone

**Figure 5:**
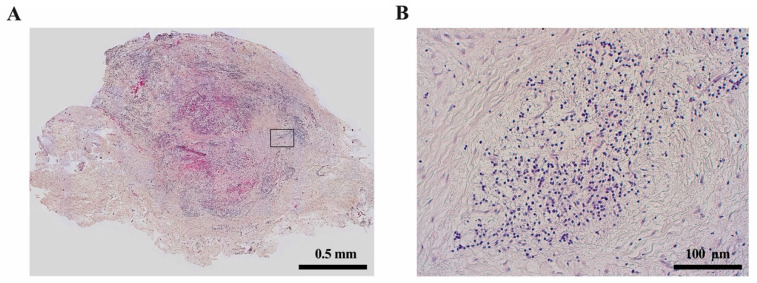
Apical granuloma developed after pulp necrosis (own collection). (**A**) H&E staining of granulation tissue with (**B**) inflammatory cell infiltrates.

Periapical lesions are considered an immunological defense reaction of the host to prevent the spread of bacterial infections from the root canal to the surrounding tissues [136]; most commonly, they present as apical granulomas (Figure 5). Most immune cells in periapical lesions are lymphocytes and macrophages [137]. Microbial components such as lipopolysaccharides get in contact with antigen-presenting cells (APC) like macrophages in the periapical tissue and induce the production of pro- or anti-inflammatory cytokines [136]. It is shown that proinflammatory cytokines like IL-1 and IL-6 can act as growth factors for ERM cells and may therefore promote radicular cyst formation [16].

It is accepted that immunologic pathways contribute to the formation of radicular cysts in periapical lesions. However, the host defense processes in apical periodontitis are still a matter of research. In radicular cysts, macrophages showed a significantly higher degree of M1-like pro-inflammatory polarization compared to apical granulomas [138]. These cells might interact with ERM cells via cytokines and growth factors and promote their proliferation. In addition to macrophages, further immunological differences between radicular cysts and apical granulomas include the expression of human leukocyte antigen (HLA)-DR, CD83, macrophage colony-stimulating factor (MCSF) and Gal3, which appears to be significantly higher in radicular cysts than in apical granulomas [139]. The infiltration of CD4- and CD8-positive T cells and the CD4/CD8 ratio seems to not differ between apical granulomas and radicular cysts according to some studies [139,140], whereas others describe an increased CD8 infiltration in radicular cysts compared to apical granulomas [141].

These data indicate that the development of apical periodontitis towards apical granulomas or radicular cysts could be controlled immunologically, e.g., by changes in macrophage polarization. Radicular cyst formation was shown to be associated with an increased M1-like proinflammatory polarization of infiltrating macrophages [138]. Increased inflammatory activity may promote the formation of radicular cysts and increased bone resorption. Therefore, the use of root filling materials with anti-inflammatory properties like mineral trioxide aggregate (MTA) may counteract the development of radicular cysts and should be analyzed further in preclinical studies [139].

## 4. Resolution of Inflammatory Responses

### 4.1. Healing of the Dental Pulp

Although the immune response of the dental pulp exerts a variety of mechanisms to protect the soft connective tissues, extensive damage cannot be restored. Whereas proteases enable immune cells to passage through, they also dissolve the extracellular matrix, and immune cells not only harm invading microorganisms but also neighboring cells. Intense and prolonged stimulation results in chronic inflammation, premature aging and reduced defense mechanisms [54,96], or in tissue degradation, which enables bacteria to populate the root canal system and migrate into the periradicular tissues via the apical foramen.

Pathogen removal by therapeutic intervention can result in the resolution of inflammation, the elimination of remaining toxins, the secretion of anti-inflammatory signals and the production of tertiary dentine [6]. Apparently, the depth of the carious lesion is a critical factor, where a full host response is observed in lesions where the remaining dentine layer is less than 0.5 mm [142]. Furthermore, the progression rate plays a role, where rapidly spreading lesions are characterized not only by a different consistency and color but also by a differing microbiota [143]. In slowly progressing lesions, mineral deposition can detain invading bacteria and restrict tissue damage [6].

However, there is a close link between inflammation and repair, which was discovered with the observation that corticosteroids could compromise healing after myocardial infarction [144], and many proinflammatory mediators in pulpal inflammation can have differential effects [145], depending on their concentration. Compounds such as TGF-β and TNF-α, but also bacterial components can promote processes of repair at low concentrations, whereas they cause detrimental effects at higher levels. In addition, stem cell differentiation may be controlled by various proinflammatory mediators [27].

Not only the initial inflammatory response but also the reparative phase is characterized by the migration of various immune cells. In addition, nerve fiber sprouting beneath the site of injury [103] is guided by pulp fibroblasts by means of complement activation and secretion of brain-derived neurotrophic factor (BDNF), which enhances the outgrowth of neurites [52]. Other neurotrophic factors such as SP, VIP, CGRP and NPY may also play a role during regenerative processes as they promote angiogenesis and stimulate the deposition of tertiary dentine [146,147]. Both nerve growth factor (NGF) and BDNF are expressed in pulp cells; they have been suggested to enhance odontoblast differentiation and thus dentinogenesis [148,149,150].

Nevertheless, depending on the intensity of the stimulus and degree of damage to the pulp, repair and healing may not be possible, and chronic inflammation and eventually pulp necrosis may be the long-term consequence.

### 4.2. Healing of the Periradicular Bone

As microorganisms, which enter the periradicular bone via the apical foramen, cause an inflammatory response and subsequently a bony lesion, this process needs to be reverted in order to achieve healing. During endodontic treatment, antibacterial strategies aim at the elimination or at least drastic reduction of microorganisms within the root canal system in order to enable healing, which is then no longer hindered by infection. The process of healing begins with the inflammation and is resolved by the clearance of the immunogen/pathogen that induces the tissue response [151]. Thus, the integrity and regular function of the periradicular bone can be reestablished. Chronic or systemic conditions negatively affect the healing potential. Diabetes mellitus may be a modulating factor of endodontic infections and may compromise the healing process of periapical tissues [152]. Hyperglycemia elevates the levels of systemic inflammatory markers [153] and alters the various functions of the immune system [154,155]. In diabetic rats, the presence of endodontic infection and periapical lesions leads to increased numbers of neutrophils, lymphocytes and leukocytes, and the level of bone resorption in endodontic lesions was greater in diabetic compared to normoglycemic rats [156].

### 4.3. Stem cells in Repair and Regeneration

An important cell source during regular tissue turnover, but also during repair is the pool of resident stem cells within the dental pulp. Mesenchymal dental pulp stem cells can be harvested from permanent teeth [157] as well as deciduous teeth [158], furthermore from the apical papilla of immature teeth with incomplete root formation [159]. Stem cells in the dental pulp are located in the perivascular niche [160] and remain quiescent until they are recruited to the site of injury upon chemotactic signaling, they migrate and differentiate into a mineralizing cell type reminiscent of odontoblasts [161]. However, pulp stem cells also express TLRs and are capable of pathogen recognition [22], but may also be recruited after activation by macrophages [162].

Whereas carious lesions are the most common cause for inflammatory reactions, traumatic impact, for example, after crown fractures, may also expose the pulp to the oral cavity and thus enable microorganisms to access the pulp chamber. In the latter case, a healthy pulp can withstand bacterial invasion for several days. Animal studies in monkeys demonstrated that the inflammatory zone did not extend more than 2 mm into the pulpal tissue even after one week of exposure to the oral cavity [163], which highlights once more the remarkable ability of this tissue to withstand a bacterial attack.

## 5. The Link between Inflammation and Malignant Transformation

Escape from the immune system is one of the “Hallmarks of Cancer” and is necessary for the establishment and spread of solid tumors [164,165]. The connection between the immune system and tumor growth is outlined by the clinical success of checkpoint inhibitors. These are antibodies that block the inhibition of signaling pathways of the immune system like the programmed cell death 1 (PD1) pathway and thus “release brakes” of the immune system and therefore promote the physiologic host defense against malignant cells. Immune checkpoints modulate the crosstalk between different immune cells like T cells and antigen-presenting cells (APC), e.g., macrophages, but also between tumor cells and the effector T cells armed to destroy the tumor cells. It is known that the incidence of malignancies and their aggressiveness are increased in immunocompromised individuals [166,167,168]. This underlines the role of the immune system in the development and progression of cancer in general.

**Figure 6:**
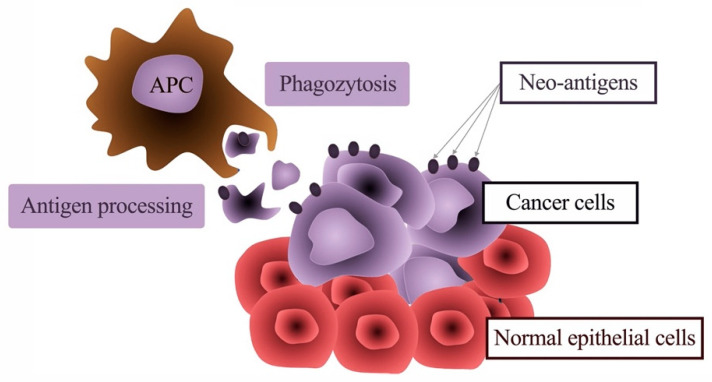
Immunological recognition of neoantigens in dysplastic lesions and tumors. Tumor cells, as well as highly dysplastic cells, express neoantigens that can be recognized by immune cells, such as professional antigen-presenting cells (APC). The tumor cells can then be phagocytized, and the antigens are processed and finally presented on major histocompatibility complex (MHC) molecules on T cells. This can initiate an immune reaction against dysplastic cells or cancer cells and therefore prevent the clinical appearance of a cancer disease.

Interestingly, neither pulpitis nor apical granuloma as a consequence of caries develops towards cancerous lesions. Most malignancies of the oral cavity arise on the basis of the oral epithelium. According to the classical understanding, oral cancer (oral squamous cell carcinoma; OSCC) is caused by the accumulation of chemical cell damage, which leads to genetic aberrations of oral epithelial cells [169,170]. The main chemical noxious agents in the western world are alcohol consumption and smoking. The genetic damage leads to an increasing disturbance in the growth regulation of the oral mucosa cells, which finally results in a malignant epithelial cell clone with the ability for unregulated proliferation and invasion [169,170]. This process is called multistep carcinogenesis [169]. Due to the genetic aberrations that result from this process, numerous altered proteins also occur in the malignant cell. These altered proteins should actually serve as neoantigens for the immune system, which should be able to distinguish and attack the malignant cell clone from healthy body cells (Figure 6) [171]. This immune response should, in principle, allow early destruction of the tumor before it becomes clinically evident.

However, the fact that oral cancers become clinically apparent shows that the immunological clearance of the tumor was not successful in these cases [172,173]. Still, we do not know the number of cases that do not become apparent because of immune surveillance. A decisive reason for this is that malignant tumors can elude the access of the immune system. Various mechanisms for this “tumor immune escape” are discussed [172,173]. One possibility is that immune cells are not able to infiltrate the tumor sufficiently. Furthermore, immune cells that infiltrate the tumor can be inactivated. One important pathway for the inactivation of immune cells is immunosuppressive checkpoints like the PD1-pathway. Another possibility is a disturbed communication between the innate and adaptive (acquired) immune system in the “immunological synapse” in the lymph node. Furthermore, an increasingly growing amount of “tumor antigen” could induce a mechanism of peripheral immune tolerance. Or, mutations that are not tumor driver-mutations can lead to neoantigens, and the tumor cells carrying these mutations get destroyed. However, if the pattern is heterogeneously expressed, not all tumor cells will carry these mutations, which will lead to an outgrowth of mutation-negative tumor cells. This selection process is comparable to the development of antibiotic resistance in bacteria.

The relevance of immunological markers has been shown for many malignancies [174,175,176,177]. As well as for other types of cancer, there is increasing evidence for an immunological influence on tumor development and progression also in oral cancer. An association of immune cells such as macrophages and T cells with the progression of oral cancer is evident [173,178,179]. However, there is increasing evidence that the development of oral cancer may also be an immunologically modulated process.

Up to two-thirds of oral cancers develop from oral leukoplakia (OLP) [180], which is, in many cases, clinically apparent years before malignant transformation [181]. Therefore, there is an urgent need to identify OLP with a high risk of malignant transformation. The gold standard for this assessment is the histologic analysis of dysplasia (D0–D3) in incision biopsies. However, the microscopic analysis is poorly reproducible between different observers [182,183]. Moreover, in many cases, OLP does not proceed, as the degree of dysplasia would indicate. It has been reported that 0–3% of hyperplasia (D0) and up to 30% of mildly dysplastic OLP (D1) [184,185] proceed to oral cancer.

Recently it has been shown that immunologic changes in the oral mucosa already precede the malignant transformation of OLP to OSCC. In OLP with malignant transformation within five years, oral mucosa macrophage cell density, as well as the proportion of immunosuppressive M2-like macrophages, was significantly increased compared to OLP without malignant transformation. These data indicate that macrophage infiltration and -polarization could be clinically used as predictors of malignant transformation in the oral mucosa.

M2-like macrophages release anti-inflammatory cytokines, inhibit T cells and show reduced antigen presentation [186,187]. This might contribute to an impaired immune response against dysplastic cells in OLP. Additionally, M2-like immunosuppressive macrophages produce growth factors that could promote malignant transformation [186]. Therefore, increased infiltration by M2-like immunosuppressive macrophages in the oral epithelium contributes to malignant transformation. However, these cells could also serve as therapeutic targets to prevent the progression of OLP to OSCC. The value of macrophages as part of an immunoscore to predict the malignant transformation of OLP is currently investigated in a multicenter prospective study (NCT03975322).

Immune modulatory treatment concepts for precursor lesions and early-stage malignancies like bladder cancer or superficial basal cell carcinomas (BCC) of the skin are already used clinically. In non-muscle-invasive bladder cancer, intravesical instillation of Bacille Calmette–Guerin (BCG), an attenuated form of Mycobacterium bovis, is used to prevent the development of invasive cancer [188,189]. The mechanism of action of BCG is not completely understood, but modulation of APCs like macrophages is proven [190]. Superficial BCC skin cancer can be successfully treated solely by topical application of Imiquimod, an agonist of the Toll-like receptor (TLR) [190]. TLR activation causes local inflammation and therefore activates immune cells and induces proinflammatory M1-like macrophages [191]. This indicates that local immunotherapy could also be an option in premalignant lesions of the oral epithelium.

In established OSCC, immunotherapy with checkpoint inhibitors is applied in cases where curative surgical and radio-oncological treatment approaches are not possible [192]. However, the first-line treatment of OSCC is the surgical resection of the primary tumor with a free resection margin of at least 5 mm [193]. The resulting defect should be reconstructed simultaneously using microvascular tissue transfer if necessary. Additionally, an elective neck dissection should be performed even if there is no radiologic evidence of lymph node metastases [193]. There is evidence that this lymph node management concept increases survival. After surgery, high-risk OSCC cases are treated with adjuvant radio- or radio-chemotherapy. Large tumors with infiltration of the skull base or prevertebral fascia without the option of surgical R0 resection can be treated with definitive radio-chemotherapy alone [193].

An interesting aspect is the question of primary malignancies of the dental pulp. As every dividing cell population of the human body is believed to be able to potentially undergo malignant transformation, the absence of primary dental malignancies appears astonishing. The dental pulp is rich in mesenchymal stem cells, and it has been shown that these cells promote tumor progression and metastatic spread [194]. Additionally, dental pulp stem cells can differentiate into several cranial neural crest cell populations like odontoblasts, osteoblasts, muscle cells and melanocytes [195]. Searching PubMed, there was only one review available addressing possible reasons for the lack of primary dental cancer [196]. The authors hypothesize that tumor growth in the restricted space of the dental pulp will lead to hemorrhage or vascular compression. Additionally, secondary dentine formation may lead to compression and pulp necrosis [196], which is treated either by root canal treatment or tooth extraction [196]. The incomplete understanding of the absence of primary dental malignancies suggests that histologic analyses of pulpectomy samples with regard to markers of malignant transformation may be promising.

## Abbreviations

3-NT	3-nitrotyrosine
BCC	basal cell carcinoma
BCG	Bacille Calmette–Guerin
BD	beta-defensins
BDNF	brain-derived neurotrophic factor
bFGF	basic fibroblast growth factor
BH4	tetrahydrobiopterin
BMP	bone morphogenetic protein
CCL	chemokine (C-C motif) ligand
CD	cluster of differentiation
CGRP	calcitonin gene-related peptide
CT	calcitonin
CXCL	chemokine (C-X-C motif) ligand
DC	dendritic cell
EGF	epidermal growth factor
ERM	epithelial cell rests of Malassez
HDP	host defense peptides
HERS	Hertwig’s epithelial root sheath
HLA	human leukocyte antigen
IFN	interferon
IL	interleukin
LBP	lipopolysaccharide-binding protein
LPS	lipopolysaccharide
LTA	lipoteichoic acid
MCSF	macrophage colony-stimulating factor
MAC	membrane attack complex
MHC	major histocompatibility complex
MPK	mitogen-activated protein kinase
NF-κB	nuclear factor-κB
NGF	nerve growth factor
NK	natural killer
NLR	NOD-like receptors
NO	nitric oxide
NOS	nitric oxide synthase
NPY	neuropeptide Y
OLP	oral leukoplakia
OONO-	peroxynitrite
OSCC	oral squamous cell carcinoma
PAMP	pathogen-associated molecular patterns
PDGF	platelet-derived growth factor
PIGF	placenta growth factor
PRR	pattern-recognition receptors
RNS	reactive nitrogen species
sGC	soluble guanylyl cyclase
SP	substance P
TGF	transforming growth factor
TLR	Toll-like receptors
TNF	tumor-necrosis-factor
Treg	regulatory T cell
VEGF	vascular endothelial growth factor
VIP	vasoactive intestinal polypeptide
